# Changing Histopathological Diagnostics by Genome-Based Tumor Classification

**DOI:** 10.3390/genes5020444

**Published:** 2014-05-28

**Authors:** Michael Kloth, Reinhard Buettner

**Affiliations:** 1 Institute of Pathology, University Hospital Cologne, Kerpener Str. 62, Cologne D-50937, Germany; E-Mail: reinhard.buettner@uk-koeln.de; 2 Center for Integrated Oncology Cologne-Bonn, Cologne D-50937, Germany

**Keywords:** human genome project, cancer genomics, tumor classification, targeted therapies

## Abstract

Traditionally, tumors are classified by histopathological criteria, *i.e.*, based on their specific morphological appearances. Consequently, current therapeutic decisions in oncology are strongly influenced by histology rather than underlying molecular or genomic aberrations. The increase of information on molecular changes however, enabled by the Human Genome Project and the International Cancer Genome Consortium as well as the manifold advances in molecular biology and high-throughput sequencing techniques, inaugurated the integration of genomic information into disease classification. Furthermore, in some cases it became evident that former classifications needed major revision and adaption. Such adaptations are often required by understanding the pathogenesis of a disease from a specific molecular alteration, using this molecular driver for targeted and highly effective therapies. Altogether, reclassifications should lead to higher information content of the underlying diagnoses, reflecting their molecular pathogenesis and resulting in optimized and individual therapeutic decisions. The objective of this article is to summarize some particularly important examples of genome-based classification approaches and associated therapeutic concepts. In addition to reviewing disease specific markers, we focus on potentially therapeutic or predictive markers and the relevance of molecular diagnostics in disease monitoring.

## 1. Approaches to a Genome-Based Tumor Classification

### 1.1. The 2008 WHO Classification of Hematological Malignancies

The 2008 WHO classification of chronic myeloid malignancies is at present the most evolved approach to a taxonomy considering defined molecular aberrations [1]. The malignancies that are included are now classified into five categories:

(1) Acute myeloid leukemia (AML) and related precursor neoplasms;
(2) Myelodysplastic syndromes (MDS);
(3) Myeloproliferative neoplasms (MPN);
(4) Myelodysplastic/Myeloproliferative neoplasms (MDS/MPN);
(5) Myeloid and lymphoid neoplasms with eosinophilia and abnormalities of *PDGFRA*, *PDGFRB*, or *FGFR1*.

**Figure 1:**
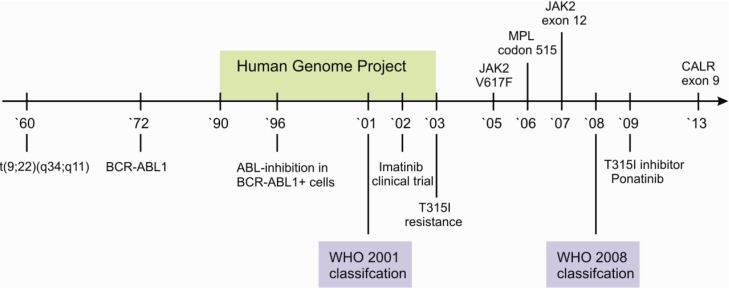
Timeline of the elucidation of genomic alterations in myeloproliferative neoplasms. Major breakthroughs in the understanding of the Ph+ neoplasm CML are depicted below the line, those in the understanding of Ph- neoplasms above. Note the significant impact of the human genome project on the elucidation on Ph- specific genomic alterations.

The integration of histology and genetics is particularly visible in the category of myeloproliferative neoplasms (MPN). Classification of specific entities into MPN is dependent on presence or absence of *BCR-ABL1*, the disease-causing translocation in CML [2]. The first description of the associated karyotype t(9;22)(q34;q11), according to an abnormally short chromosome 22, was published as early as 1960 and is widely known as the Philadelphia (Ph) chromosome [3]. Due to the high specificity of *BCR-ABL1*, its detection is mandatory for diagnosis of CML and is further underscored by the influence on therapy with the small molecule tyrosine kinase inhibitor (TKI) Imatinib [4]. Nevertheless, the remarkable journey, from the genomic aberration to the specific therapy, required approximately forty years and started long before the elucidation of the human genome (Figure 1).

Further myeloproliferative neoplasms are characterized by the absence of *BCR-ABL1*, including the three Ph-negative classic MPNs—polycythemia vera (PV), essential thrombocythemia (ET) and progressive myelofibrosis (PMF). Despite the absence of *BCR-ABL1*, it has consecutively been shown that these MPNs are themselves also characterized by additional recurrent aberrations [5,6] (Figure 1).

**Figure 2:**
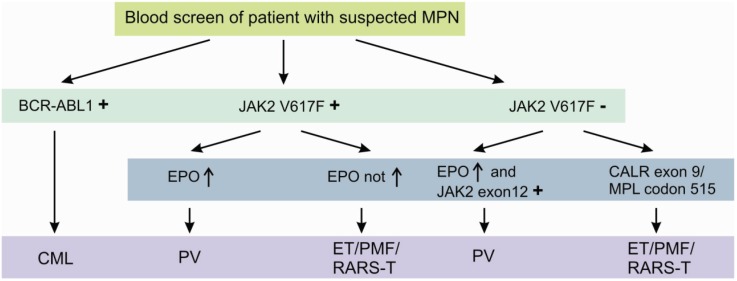
Diagnostic algorithm of classic myeloproliferative neoplasms using specific molecular aberrations. Detection of the molecular aberrations depicted above is highly suggestive for the suspected myeloproliferative disorder. Nevertheless, at least in the case of absence of these specific aberrations, a bone marrow biopsy should be performed.

The *JAK2 V617F* mutation is detectable in approximately 95% of all PV patients and 50% of both ET and PMF patients [7,8,9]. It is further detected in patients with refractory anemia with ring sideroblasts and thrombocytosis (RARS-T), but in less than 5% of patients with acute myeloid leukemia (AML) or myeloid dysplastic syndrome (MDS) and not in solid tumors [10,11]. Although, the diagnostic process is initially dominated by peripheral blood cell count and serum erythropoetin (EPO) levels, *JAK2 V617F* or less common *JAK2 Exon 12* mutations [12] confirm the diagnosis of a suspected PV without the need of a bone marrow biopsy [13]. Beside the two most important genetic aberrations in the diagnostic algorithm of MPNs, namely *BCR-ABL1* and *JAK2 V617F*, several other potentially helpful recurrent aberrations are known. These include the presence of recurrent mutations in *CALR exon 9* [14] and *MPL codon 515* [15], essentially occurring in *JAK2 V617F* negative cases (Figure 2).

Another important example of the integration of molecular aberrations in the 2008 WHO classification of hematological malignancies is the newly introduced group of myeloid and lymphoid neoplasms with eosinophilia and abnormalities of *PDGFRA*, *PDGFRB*, or *FGFR1*. This reclassification highlights the consideration of the targetable alterations *FIP1L1-PDGFRA* or *PDGFRB*-rearrangements and those harboring *FGFR1*-rearrangements, indicating response or resistance to Imatinib [2,16].

### 1.2. Lung Cancer as a Paradigm: Advances in the Molecular Characterization of Solid Malignancies

The ongoing comprehensive characterizations of solid tumors, such as those conducted by The Cancer Genome Atlas (TCGA) will significantly impact upcoming tumor classifications. This also includes lung cancer, the leading cause of cancer death worldwide [17] and an example for substantial advances by genome-based therapy approaches [18]. In general, NSCLC is subclassified into adenocarcinoma, squamous cell carcinoma and large cell carcinoma. Future classifications need to characterize clinically relevant subtypes, instead of the traditional distinction of non-small cell lung cancers (NSCLC) and small-cell lung cancer (SSLC). Concepts for a reclassification of lung adenocarcinoma were suggested, particularly with respect to reclassify large cell carcinoma based on genomic aberrations into adenocarcinoma, squamous cell carcinoma and large cell neuroendocrine carcinoma, respectively. Recommendations for (immuno)histological diagnostic work-up, and also for determining specific molecular aberrations in lung adenocarcinoma subtypes have been published [19].

Major rationales for changes in adenocarcinoma histologic variants are that the invasive mucinous type shows frequent mutations in *KRAS* and hardly any in *EGFR*, whereas non-mucinous adenocarcinoma of predominant lepidic subtype is characterized by frequent mutations in *EGFR* and fewer in *KRAS* [20]. In this context, it is worth noting and described in more detail below, that clinical studies with the TKIs Gefitinib and Erlotinib showed significantly improved survival in patients suffering from lung adenocarcinoma harboring mutations in the kinase domain of *EGFR* [21,22]. Besides the mutations in *EGFR* and *KRAS*, the targetable translocation *EML4-ALK* recurrently occurs in lung adenocarcinoma [23] and is most frequent in tumours with mucinous signet-ring appearance. Clinical trials using the tyrosine kinase inhibitors Crizotinib and Ceritinib have now shown improved progression-free survival in those patients [24,25].

In contrast, squamous cell carcinoma of the lung is commonly associated with mutations in the *NFE2L2/KEAP*-axis [26] and often harbors targetable alterations of *FGFR1* [27] and in fewer cases mutations in *DDR2*. Interestingly, DDR2-transformed cell lines maintain SRC phosphorylation and are sensitive to Dasatinib [28], proved by the response in squamous cell lung cancer patients [29].

Beyond the combined loss of *RB1 and TP53* in neuroendocrine pulmonary tumors [30], it was shown that low- and intermediate-grade pulmonary cacinoids harbor recurrent mutations in chromatin remodeling complexes [31], whereas the high-grade neuroendocrine tumor, small cell lung cancer, is associated with sequential changes, including the deletion of *PTEN* [32].

**Figure 3:**
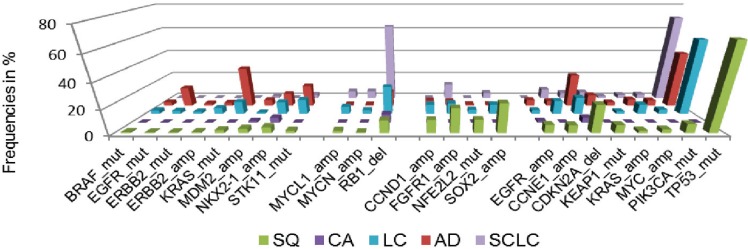
Frequencies of significant genomic alterations in histological subgroups of lung cancer. Colors of histological subtypes are encoded as follows: green—squamous cell lung cancer (SQ), purple—carcinoid tumor, light blue—large cell lung cancer (LC), red—adenocarcinoma of the lung (AD), dark blue—small cell lung cancer (SCLC). Data adapted from [33].

Additionally, we assessed cancer genome alterations linked to histomorphological and immunohistochemical features, considering high therapeutic relevance and improved patient outcome [33] (Figure 3). By this approach, we devised a genomic-based prediction model of lung cancer subtypes. This model shows that the majority of large cell cancers could be reassigned to adenocarcinoma, squamous cell carcinoma or small-cell lung cancer. By the combined analysis of immunohistochemical, genomic and clinical features it becomes further evident that personalized approaches significantly improve the outcome of patients with advanced lung cancer and other solid cancers.

Beyond the principally well-characterized situation in lung cancer, we already know of highly recurrent genomic alterations in many other solid malignancies. Prostate cancer is the most common cancer in men and is characterized by fewer mutations in typical cancer genes, when compared to other solid cancers [34,35,36]. However, genomic alterations in androgen signaling, the rearrangement of *ETS* transcription factors, especially the fusion of *TMPRSS2-ERG* [37], as well as the deletion of *PTEN* [38] are known to be highly recurrent in primary cancers [39], early-onset cancers [40] and castration-resistant prostate cancers [41]. Beside the outstanding therapeutic role of androgen deprivation, recent efforts investigate a mechanistic rationale of PARP inhibition in *ETS*-rearranged prostate cancers [42].

### 1.3. EWS and the Importance of Translocations in the Diagnostic Workup of Mesenchymal Malignancies

Tumors ascribed to the family of Ewing’s sarcomas or primitive neuroectodermal tumors (PNET) are the second most common bone tumors in children, but can also arise from any other tissue. The recent molecular understanding of these aggressive tumors has greatly advanced new therapeutic approaches. However, whereas chemotherapy improved the survival rate from 10% to 70%–80% in localized disease, the survival of patients with distant metastases is still poor [43]. The WHO classification of Ewing sarcoma (ES) as a single entity is underlined by its cytogenetic signature t(11;12)(q24;q12), according to the translocation *EWSR1-FLI1* in approximately 85% of patients. Although, *EWSR1* translocations can be found in many other mesenchymal tumors, almost all of the remaining ES cases are characterized by further ES-specific *EWSR1* translocations [44,45]. This also includes the second most common cytogenetic aberration t(21;22)(q22;q12), corresponding to *EWSR1-ERG* [46] (Table 1).

Intriguingly, the well-characterized molecular situation of ES is in contrast to the fact that the cell of origin of the small round cell appearing tumor is not known. A further complication of the histological diagnosis is caused by atypical ES, including large/epithelioid/clear/spindle cell ES, vascular-like ES, adamantinoma-like ES, ES with neuroectodermal features, synovial sarcoma-like PNET and sclerosing PNET [47]. Beyond this difficult histopathological classification, sarcomas may be genetically classified from a near-diploid karyotpe to a more complex genomic instability. The first is characterized by highly recurrent translocations, the latter by numerical and structural abnormalities affecting multiple chromosomes [48]. In the case of small round cell appearing sarcoma, recently, new subtypes were defined by recurrent translocations of BCOR-CCNB3 [49] and CIC-DUX4 [50].

Furthermore, we already know several diagnostically relevant genomic rearrangements in soft tissue malignancies [51]. The recurrently occurring reciprocal translocation t(X;18) is characteristic of synovial sarcoma, and leads to the potentially therapeutic relevant SSX-S18 protein [52]. The translocation *FUS-CHOP* is detected in myxoid liposarcoma [53] and recurrent amplifications of the E3-Ubiqutitin ligase *MDM2*, as well as *CDK4*, in well differentiated and dedifferentiated liposarcoma [54,55]. Interestingly, ongoing efforts investigate the reactivation of p53 by MDM2-p53 interaction inhibitors [56].

**Table 1:** Fusion partners of *EWSR1*-rearrangements in different soft tissue tumors. Overlapping rearrangements in different histopathological entities are in bold. Adapted from [44,45].

Histological type	Translocation	EWS-rearrangements
Ewing’s Sarcoma	t(11;22)(q24;q12)	EWSR1-FLI1
	t(21;22)(q22;q12)	EWSR1-ERG
	t(7;22)(q22;q12)	EWSR1-ETV1
	t(17;22)(q21;q12)	EWSR1-ETV4
	t(2;22)(q36;q12)	EWSR1-FEV
	inv(22)(q12q12)	EWSR1-PATZ1
	t(2;22)(q31;q12)	EWSR1-SP3
	t(20;22)(q13;q12)	EWSR1-NFATC2
	t(4;22)(q31;12)	EWSR1-SMARCA5
	t(17;22)(q12;q12)	EWSR1-E1AF
	inv(22)(q21;12)	EWSR1-ZSG
Angiomatoid fibrous histiocytoma	t(12;22)(q13;q12)	**EWSR1-ATF1**
	t(2;22)(q33;q12)	**EWSR1-CREB1**
Clear cell sarcoma	t(12;22)(q13;q12)	**EWSR1-ATF1**
	t(2;22)(q33;q12)	**EWSR1-CREB1**
Malignant gastrointestinal neuroectodermal tumor	t(12;22)(q13;q12)	**EWSR1-ATF1**
	t(2;22)(q33;q12)	**EWSR1-CREB1**
Myoepithelial tumor of soft tissue and bone	t(1;22)(q23;q12)	EWSR1-PBX1
	t(19;22)(q13;q12)	EWSR1-ZNF444
	t(6;22)(p21;q12)	EWSR1-POU5F1
Extraskeletal myxoid chondrosarcoma	t(9;22)(q22;q12)	EWSR1-NR4A3
Myxoid liposarcoma	t(12;22)(q13;q12)	EWSR1-DD1T3

### 1.4. Cancer of Unknown Primary Origin (CUP)

Carcinoma of an unknown primary origin (CUP) is descriptive of a metastatic cancer without an identifiable primary tumor site. CUP accounts for 3%–5% of all cancer diagnoses and is usually characterized by an aggressive metastatic growth and a challenging clinical presentation. In theory, CUP could be considered as a unique biological entity, or in the opposite view, as a group of different entities. The classification of CUP is essentially based on the prognostic outcome, thereby distinguishing favorable and unfavorable carcinomas [57,58,59] (Table 2).

**Table 2:** Classification of cancers of unknown primary origin.

Clinically favorable CUP	Clinically unfavorable CUP
Extragonadal germ-cell cancer	Metastatic adenocarcinoma
Peritoneal papillary adenocarcinoma	Non papillary malignant ascites
Adenocarcinoma in axillary lymph nodes	Multiple cerebral metastases
Cervical squamous-cell carcinoma	Squamous-cell carcinoma of the abdominopelvic cavity
Neuroendocrine carcinoma	Lytic bone metastases
Blastic bone metastases and PSA elevation	

This classification predominantly depends on the morphological and immunohistochemical appearance. At present, the specimen is investigated by the use of specific antibodies (investigated epitopes in parentheses):

(1) Identification of the cancer type:
  Carcinoma CK AE1/3), mesothelioma (Calretinin, BerEP4), sarcoma (Vimentin), lymphoma (LCA), melanoma (HMB-45, MITF, S100);
(2) Identification of the subtype:
  Adenocarcinoma CK7, CK20), squamous cell carcinoma (CK5/6, p40, p63), hepatocellular carcinoma (Hepar1), renal cell carcinoma (RCC, PAX8, CA9), urothelial carcinoma (GATA3, S100P, Uroplakin), thyroid cancer (hTG, TTF1), neuroendocrine cancer (CD56, Synaptophysin, ChromoA), germ-cell tumor (PLAP);
(3) Identification of the origin:
  Lung TTF1, NapsinA), colorectal cancer (CDX2, CK20), breast (ER, PR), pancreas (CDX2, CK7, CK20), ovary (Ca125, ER, WT1), prostate (PSA, PSAP, AR).

However, only 30% of all cases studied can be assigned to a primary tumor and even fewer benefit from a change in therapy regimen. Over the last decade, the molecular characterization by gene-expression profiling in various tumor types has led to the development of several gene signature assays with identification rates of a putative tissue of origin in up to 90% of cases. Furthermore, it was shown that CUP can be treated along actionable genomic alterations and recent whole exome sequencing revealed the existence of known recurrent mutations [60]. These include therapeutically relevant mutations in *PIK3CA*, *MET*, *FGFR3*, *IDH1* as well as several others and it has been already demonstrated that targeted therapies can significantly influence a more favorable outcome in CUP patients [61]. As another variation on this theme, a drug-sensitizing genome alteration in one tumor type may not confer drug susceptibility in another histology, as has been observed in the case of *BRAF* mutations that confer *MEK* and *BRAF* dependency in melanomas [62,63] but not in colorectal carcinomas resulting from *EGFR* activation [64]. These interactions highlight the need for classifications integrating cancer genome alterations, but also histomorphological and immunohistochemical features.

## 2. Monitoring of Malignancies

Molecular monitoring of CML is the most advanced routinely used surveillance strategy and reflects the need for standardization and quality controls of diagnostic tests. A main objective is the identification of patients, which have a worse response or resistance to therapy with tyrosine kinase inhibitors. The quantification of *BCR-ABL1* transcripts in peripheral blood is thereby of outstanding value in the early phase after initial drug administration, corresponding to the molecular response rate (MMR) upon TKI therapy [65,66,67]. The MMR is calculated in relation to a control gene (e.g., *BCR* or *ABL1*) and was standardized between laboratories using the international scale (IS) [68]. Essentially, the WHO has undertaken extensive efforts to simplify and standardize the assay by providing reference reagents. The importance of such efforts is reflected by a comparable poor overall survival of patients with an early MMR >10%, in turn leading to universally valid changes in the associated NCCN and ELN guidelines [66].

Despite significant progress in therapy of lung adenocarcinoma, all patients with *EGFR* mutations and *ALK* or *ROS1* translocations receiving specific tyrosine kinase inhibitors will ultimately experience relapse. Recent work highlights the potential of noninvasive detection and monitoring of resistance mutations in free circulating plasma DNA of lung cancer patients. The most prominent example is the known resistance mechanisms mediated by T790M in *EGFR* [69]. Beside the potential of directly targeting cancers harboring T790M by new tyrosine kinase inhibitors [70], it is important to note that T790M cells proliferate more slowly, thereby enabling resensitizing of tumors to primarily used TKIs after temporary withdrawal of the drug. Since it is difficult in clinical practice to undertake repeat biopsies at multiple metastatic sites, noninvasive targeted sequencing techniques may further enable additional therapeutic strategies [71]. It thereby becomes evident that new diagnostic techniques will not only lead to major influences in treatment and monitoring guidelines, but will influence therapeutic guidelines and also the classification of tumors. Especially, the opportunity of non-invasive testing seems to be an attractive and emerging field in diagnostic and therapeutic concepts, as further delineated by testing for *TMPRSS2-ERG* in the case of suspicion of prostate cancer [72].

## 3. Clinical Success of Targeted Therapeutic Approaches Based on Molecular Biomarkers

### 3.1. BCR-ABL1

As depicted above, *BCR-ABL1* is the driving lesion in CML, leading to the development of the first small molecule TKI, Imatinib [4,73]. Several multicenter studies confirmed that the overall survival of advanced-stage cancer patients in a chronic disease phase climbed from approximately 50% before 2002 up to approximately 90% after introduction of Imatinib in 2002 [74]. The success of the targeted approach in CML patients is further underscored by ongoing studies investigating the discontinuation of Imatinib [75] and the groundbreaking question of a potential cure [76]. Despite these promising developments, several further therapeutic possibilities for second line therapies are underway. These include the use of second generation TKIs in case of therapeutic failure or intolerance, e.g., Dasatinib, Nilotinib, Bosutinib and Ponatinib, the latter particularly used in case of a secondary resistance by T315I mutation [75] (Figure 1).

### 3.2. BRAF

The most common melanoma mutation in *BRAF exon 15*, the activating mutation V600E, leads to response rates of more than 50% of all patients treated with the specific TKI Vemurafenib. Further, therapy with Vemurafenib is associated with a relative reduction in death of 63% when compared to standard therapeutic regime with Dacarbazine [62]. In contrast to the activating biology of *BRAF V600E*, ongoing clinical trials investigate the potency of Dasatinib in tumors harboring inactivating *BRAF exon 11* mutations (NCT01514864 clinicaltrials.gov).

### 3.3. EGFR-Family and KRAS Mutational Status

**Figure 4:**
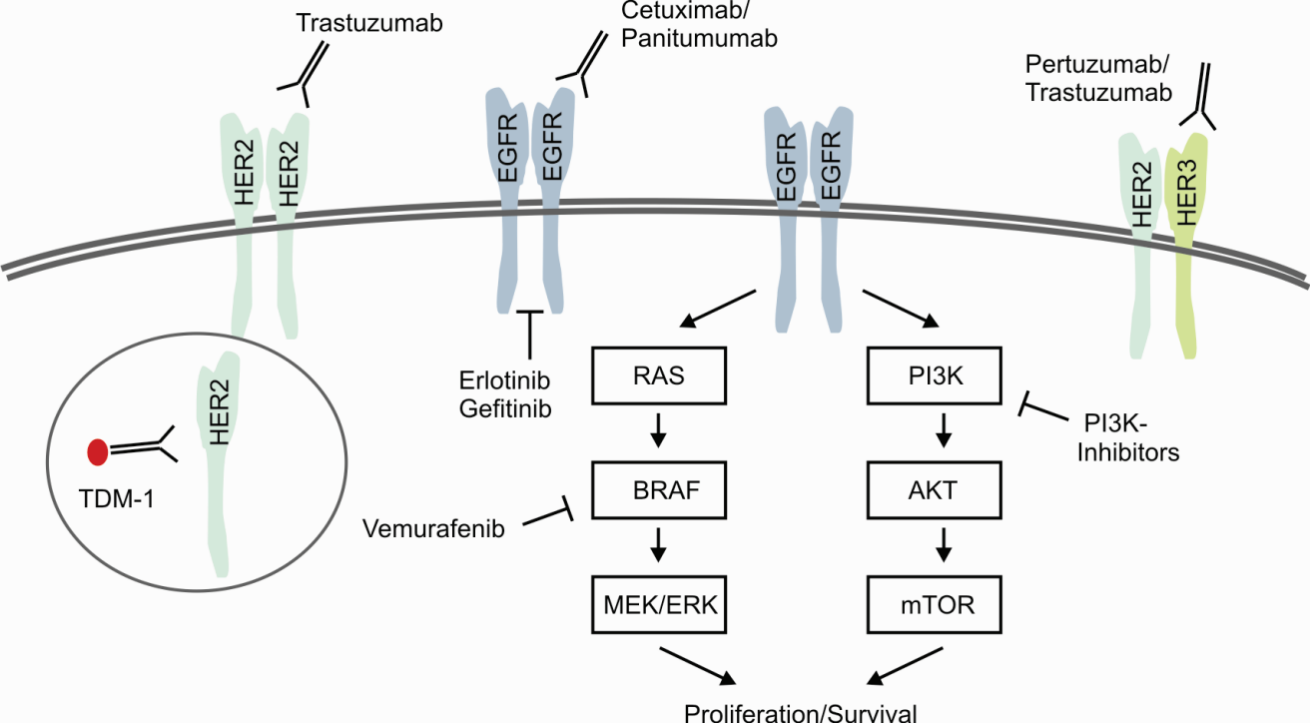
Predictive Biomarkers for Targeted and Selective Therapies. Signaling of EGFR-family receptors is characterized by homo-/heterodimerization and subsequent activation of the targetable downstream signaling pathways RAS/RAF and PI3K/AKT. Present therapeutic approaches focus on the inhibition of ligand-dependent activation, dimerization and receptor tyrosine kinases. Immunoconjugates, e.g., T-DM1, specifically deliver chemotherapeutic agents by the process of receptor internalization. As described in the text in more detail, ongoing efforts investigate the effectiveness of combined or dual approaches.

Genetic aberrations in members of the *EGFR*-family are well known for targeted therapies, including HER2- and EGFR-targeted inhibition of downstream signaling cascades. *HER2/ERBB2* is primary known as being amplified and activated in breast cancer causing high recurrence rates and increased mortality in approximately 15% of all patients [77]. Patients with amplification of *HER2/ERBB2* treated with the monoclonal antibody Trastuzumab in combination with chemotherapy showed improved outcome in several studies [78]. Beyond Trastuzumab several ongoing studies are investigating further drug therapies targeting the HER2-axis. These include the combined inhibition of HER2/HER3-heterodimerization and activation by Trastuzumab/Pertuzumab [79] and the use of the covalent immunoconjugate Trastuzumab-Emtansine (T-DM1) [80] (Figure 4). Moreover, several trials evaluate the therapeutic significance of small molecule inhibition in *HER2*-positive breast cancer, e.g., Lapatinib, Afatinib, Pazopanib and Neratinib [81].

Comparable to the situation in *HER2*-amplified breast cancer, substantial progress has been made by the introduction of EGFR-targeted therapies in the treatment of lung cancer and colorectal cancer. These efforts become evident by comparing the median overall survival of lung adenocarcinoma patients under standard therapeutic regimes of approximately 12 months with approximately 2 years under EGFR-targeted therapy with Erlotinib or Gefitinib in *EGFR*-mutated cancers [82]. As depicted above, further efforts are made to overcome primary and secondary therapeutic resistance by next generation TKIs [70]. Similar positive achievements were made for treatment of metastatic colorectal cancer (mCRC) by an improvement of survival from 12 months with fluoruracil monotherapy up to approximately 2 years with EGFR/VEGFR-targeted therapy combined with chemotherapy [83].

Beyond that, it recently became evident that we need to predict therapeutic response to cetuximab/panitumumab in mCRC not only by *KRAS* mutational status, but also by *NRAS* mutational status [84], highlighting the increasing importance of mutations in downstream or interacting pathways. As depicted above, it becomes also clear that combined approaches, like the inhibition of the EGFR/BRAF-axis in *BRAF V600E* mutated colorectal cancers [64], could be used to overcome primary resistance in histological subtypes.

## 4. Conclusions

The purpose of integrating pathogenetic and molecular information into disease classification systems, exemplified by the 2008 WHO classification of hematological malignancies, reflects the high clinical relevance for predicting therapy outcome and prognosis. The human genome project and emerging technologies in the last decade have led to fundamental pathogenetic breakthroughs, which substantially improved the translation into clinical practice and individual therapeutic possibilities. Altogether, this data underlines the significant influence of cancer genomics and the substantial increase in genomic information on the process of defining tumor entities and effective and selective treatment approaches.
